# Perspectives on the Treatment of Advanced Thyroid Cancer: Approved Therapies, Resistance Mechanisms, and Future Directions

**DOI:** 10.3389/fonc.2020.592202

**Published:** 2021-01-25

**Authors:** Ashleigh Porter, Deborah J. Wong

**Affiliations:** Division of Hematology/Oncology, Department of Medicine, Los Angeles, CA, United States

**Keywords:** thyroid cancer, tyrosine kinase inhibitor, BRAF mutation V600, mechanisms of resistance to therapy, anaplastic thyroid cancer, medullary thyroid cancer, differentiated thyroid cancer, papillary thyroid cancer

## Abstract

For differentiated thyroid cancer (DTC), systemic therapy with radioactive iodine (RAI) is utilized for radiosensitive disease, while for radioiodine refractory (RAIR) disease, current standard of care is treatment with multikinase tyrosine kinase inhibitors (TKI). For BRAF-mutant DTC or anaplastic thyroid cancer (ATC), treatment with inhibitors targeting BRAF and MEK are important advances. RET-inhibitors for RET-mutated medullary thyroid cancer (MTC) recently have been FDA-approved for metastatic disease. Nevertheless, treatment of thyroid cancer resistant to current systemic therapies remains an important area of need. Resistance mechanisms are being elucidated, and novel therapies including combinations of BRAF and MEK inhibitors with RAI or other targeted therapies or TKIs combined with checkpoint inhibition are current areas of exploration.

## Introduction

The incidence of thyroid cancer (TC) in the United States is rapidly increasing, with over 52,000 new patients diagnosed and greater than 2,000 deaths recorded each year (1). Whether this is secondary to increased frequency of cross-sectional imaging, better sensitivity in various imaging modalities or a true increase in incidence has been questioned, but regardless, prognosis is generally exceptional, with the vast majority of patients surviving at least ten years, even in the setting of locally advanced or widespread disease. The clinical course can be quite variable between different patients and among the TC subtypes.

The three main subtypes of TC include DTC, MTC, and ATC. DTC comprises 85% of all thyroid cancers, with papillary thyroid cancer (PTC) the most common histologic subtype. MTC and ATC make up 2–8 and 1% of diagnoses, respectively, generally behave more aggressively than DTCs, and can often be less responsive to therapy. While 45% of patients with metastatic DTC are alive at ten years (2), only 20% of patients with MTC are living at 10 years. Though ATC is an epithelial-derived carcinoma and often arises from DTC, prognosis is significantly worse, with a median overall survival of 3–6 months (3).

TCs are generally successfully managed with a multimodal approach, incorporating surgical resection with thyroidectomy and lymph node dissection when disease only involves local structures, followed by ablation with RAI as adjuvant therapy for patients at high risk for recurrence, and thyroid hormone suppression long-term. RAI also is utilized in the setting of iodine sensitive recurrent and metastatic disease. The National Comprehensive Cancer Network (NCCN) guidelines recommend considering other systemic therapies for progressive, disseminated disease and/or symptomatic disease that is refractory to RAI. Cytotoxic chemotherapy such as adriamycin has limited utility for metastatic TC (4); in contrast, targeted agents are the mainstay of standard therapy, building on the knowledge that aberrant signaling of the MAPK and PI3K/Akt/mTOR pathways are responsible for tumorigenesis (5). Current options for systemic therapy involve the use of TKIs targeting these aforementioned pathways which are often both effective in controlling disease and have manageable toxicity. Inevitably, however, most TCs develop resistance. Mechanisms of resistance and strategies to overcome treatment resistance are areas of active investigation.

## Pathogenesis of Thyroid Cancer

**Figure 1:**
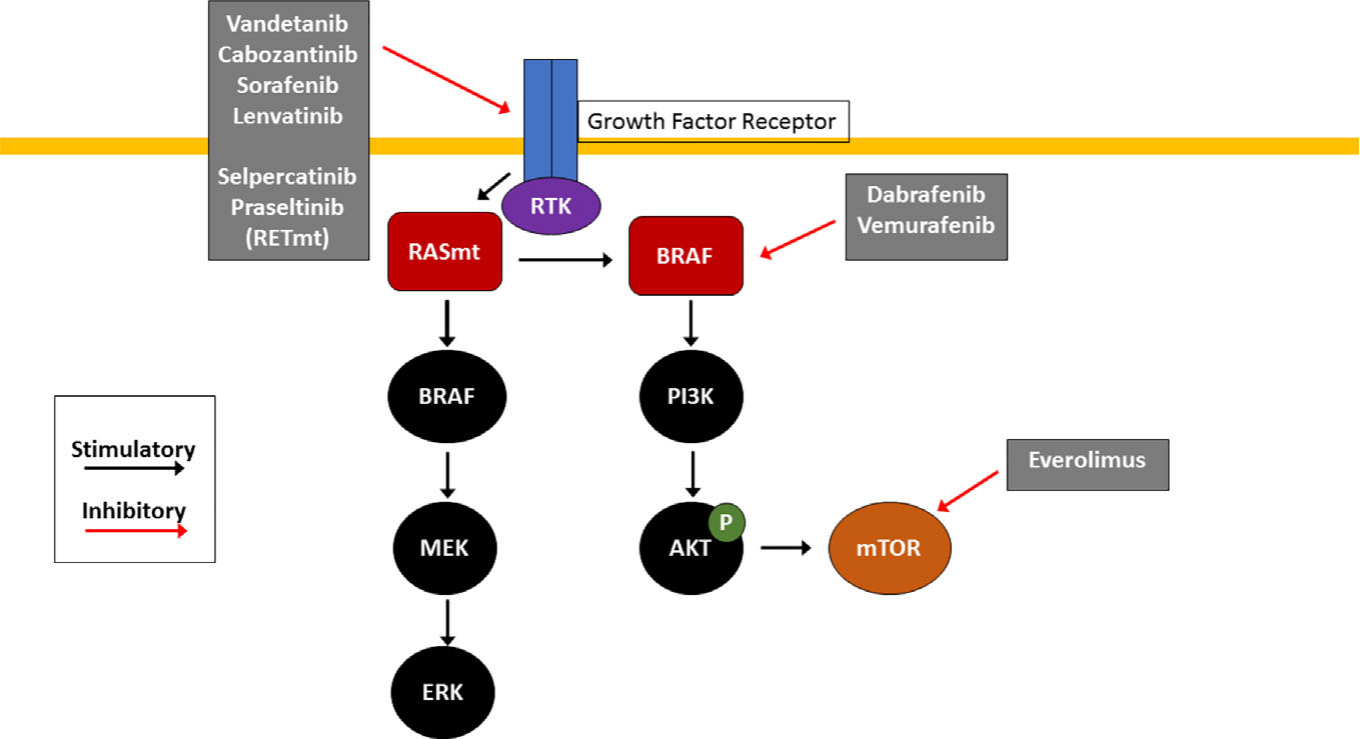
Molecular Pathogenesis of Thyroid Cancer. RAS/RAF/MEK and PI3K/AKT/mTOR pathways are key signaling pathways in thyroid cancer pathogenesis. Mutations in RAS (RASm) or BRAF result in constitutive activation of the MAPK pathway, causing downstream activation of the MAPK and PI3K pathways which promotes cell growth and tumorigenesis. Effective agents include multikinase inhibitors (cabozantinib, vandetanib, sorafenib and lenvatinib) which inhibit receptor tyrosine kinases (RTK) at the cell surface, selective RET inhibitors (selpercatinib and pralsetinib) which inhibit mutant RET RTK (RETm), BRAF V600E inhibitors (dabrafenib and trametinib) and the mTOR inhibitor everolimus. These small molecule inhibitors are used clinically for treatment of RAIR TC with the goal of arresting uncontrolled proliferation.

Like many other cancers, TC arises as a result of accumulation of multiple genetic mutations that cause abnormal cellular proliferation and prolonged survival of malignant cells. Virtually all TC pathogenesis centers around aberrant signaling involving the PI3K/Akt/mTOR and MAPK signaling pathways, which under normal circumstances, both help regulate cellular functions and survival (**Figure 1**).

The PI3K/Akt/mTOR pathway is classically activated by induction of receptor tyrosine kinase (RTK) at the cell membrane. Activated intracellular PI3K phosphorylates and activates AKT. AKT then travels inside the nucleus to upregulate several oncogenes as well the mTOR pathway, triggering tumorigenesis (5, 6).

Similar to the PI3K/Akt/mTOR pathway, MAPK signaling is stimulated first by activation of a RTK. RTK then activates multiple other genes, including RAS, BRAF, MEK, and ERK. ERK ultimately enters the nucleus to promote tumorigenesis. Most commonly mutated genes in TC include those in the MAPK pathway—BRAF and RAS mutations as well as RET fusions—which in total account for approximately 80% of cases. Notably, activation of RET, a RTK which when constitutively activated either by mutation or fusion with another partner such as PTC1 or PTC3, is involved in the pathogenesis of 5–30% of PTCs, in the vast majority of familial MTC (96% of cases) as well as in sporadic MTC (25–50% of cases), and results in constitutive activation of the MAPK signaling pathway which promotes cell growth and tumorigenesis (6, 7). RET inhibition has been an important advance for treatment of MTC, while blockade with BRAF and MEK inhibitors are a mainstay of therapy for BRAF-mutated ATC and DTC. Mutations in RET, RAS, and BRAF tend to be mutually exclusive in PTC, underscoring the importance of constitutive activation of the MAPK pathway for TC (8).

## Radioactive Iodine

Thyroid cells have the unique ability to uptake iodine from the blood. Consequently, for DTC which has retained this property, treatment with RAI, ^131^I, is a mainstay of therapy as adjuvant therapy, to address micrometastatic disease or as treatment for limited low burden metastatic disease (9). Over time, however, RAI becomes ineffective as many DTCs lose the ability to uptake iodine or as patients have received maximal lifetime doses of ^131^I. A joint statement from societies including the American Thyroid Association (ATA) lists clinical scenarios indicating RAIR disease, including (1) lack of ^131^I uptake on diagnostic ^131^I scan (2), no ^131^I uptake on a ^131^I scan performed several days after ^131^I therapy (3), selective ^131^I uptake in only some tumor foci (4), progression of metastatic DTC despite ^131^I uptake and (5) progression of metastatic DTC despite a cumulative ^131^I activity of 600mCi (9). Whether DTC can be resensitized to RAI is a subject of many research studies. As MTC and ATC do not uptake iodine, RAI is not an effective systemic therapy strategy for these subtypes.

## Multikinase Inhibitors

The use of kinase inhibitors has proven to be an effective treatment option for metastatic TC given the activity of the PI3K/Akt/mTOR and MAPK signaling pathways in this disease. Current Food and Drug Administration (FDA) approvals for kinase inhibitors in thyroid cancer include vandetanib, cabozantinib, sorafenib, and lenvatinib, which have activity against many RTKs, including the vascular endothelial growth factor (VEGF) isoforms (**Table 1**).

**Table 1:** Tyrosine Kinase Inhibitors with Activity for Thyroid Cancers.

Drug	Trial Name	Indication	Targets	PFS (months)	ORR
Vandetinib	ZETA (10)	MTC	EGF, RET, VEGF2, VEGF3	30.5 *vs* 19.5 placebo	44%
Cabozantinib	EXAM (11)	MTC	c-MET, RET, VEGF2	11.2 *vs* 4 placebo	28%
Sorafenib	DECISION (12)	DTC	VEGF1-3, PDGF, FGF, KIT, RET	10.8 *vs* 5.8 placebo	12.20%
Lenvatinib	SELECT (13)	DTC	VEGF1-3, FGF1-4, PDGF, KIT, RET	18.3 *vs* 3.6 placebo	64.80%
Dabrafenib/Trametinib	BRF117019 (14)	ATC	BRAF V600E/MEK1&2	NR	69%
Vemurafenib	NCT01286753 (15)	PTC	BRAF V600E	18.2	39%
Selpercatinib	LIBRETTO-001 (16)	MTC	RET	NR	73%
Pralsetinib	ARROW (17)	MTC	RET	NR	74%

PFS, progression free survival; ORR, overall response rate; MTC, medullary thyroid carcinoma; DTC, differentiated thyroid carcinoma; ATC, anaplastic thyroid carcinoma; PTC, papillary thyroid carcinoma; NR, not reached.

Vandetanib, a multikinase inhibitor that targets epidermal growth factor (EGF), RET, VEGF2, and VEGF3 receptors, is FDA approved for use in MTC based on results of the ZETA trial (10) which compared the effects of vandetanib dosed at 300 milligrams (mg) daily to placebo in 331 patients with advanced and unresectable MTC. Patients treated with vandetanib had a longer progression free survival (PFS) (30.5 *vs* 19.3 months); hazard ratio (HR) 0.46; 95% confidence interval (CI) 0.31 to 0.69; P < 0.001). 44% of patients achieved partial response (PR).

Subsequently, cabozantinib was approved by the FDA for advanced, progressive or symptomatic MTC. Cabozantinib works specifically by inhibiting c-MET, RET, and VEGF2 receptors. Inhibition of c-MET has been hypothesized to portend longer responses and delayed development of resistance. The EXAM trial included 330 patients with advanced MTC with progressive disease for at least 14 months (11). Patients treated with 140 mg daily of cabozantinib had a median PFS of 11.2 months compared to 4 months for placebo (HR 0.28; 95% CI 0.19–0.30; P < 0.001). 28% of patients treated with cabozantinib achieved a PR with a median duration of response of 14.7 months. Although PFS was shorter than that reported for vandetanib, this was attributed to the variability in the respective patient populations of each trial. Patients in the ZETA trial had relatively indolent disease while the EXAM trial required progressive disease for enrollment. Furthermore, cabozantinib has promising activity as frontline therapy for RAIR as a phase II single arm study of 35 patients with RAIR-DTC demonstrated 54% PR and 43% stable disease, with an 80% clinical benefit rate at six months (18). Cabozantinib is currently being evaluated in the Phase III study COSMIC-311 for patients with DTC that has progressed on up to two prior VEGFR inhibitors (NCT03690388).

Sorafenib, an inhibitor of VEGF1-3, platelet derived growth factor (PDGF), fibroblast growth factor (FGF), KIT, and RET, is approved for RAIR DTC. Benefit of treatment is modest at best, with a PFS of 10.8 months (HR 0.59; 95% CI 0.45–0.76; P < 0.0001) and an overall response rate (ORR) of 12.2%** **with no benefit in overall survival (OS) (12).

The final TKI approved for use in advanced DTC is lenvatinib, which targets VEGF1-3, FGF1-4, PDGF, KIT, and RET and is the only kinase inhibitor that has shown survival benefit. Data from the SELECT trial revealed a PFS of 18.3 months for lenvatinib *versus* 3.6 months for placebo, an ORR of 64.8% and four documented complete responses. An OS benefit was demonstrated on subgroup analysis of patients older than 65 years of age (OS not reached *vs.* 18.4 months in placebo arm); however, the validity of this benefit remains unclear and has not been reproduced in other studies (13).

## Braf Inhibition

There has been a significant amount of success in targeting BRAF driver mutations most notably in the treatment of melanoma, where approximately 50% of cases harbor activating BRAFV600E mutations. For DTC, about 40% of PTCs are BRAF-mutated (8), and 20–50% of ATCs harbor a BRAF V600 mutation. The safety and efficacy of the BRAF inhibitor dabrafenib combined with trametinib, an inhibitor of MEK1/2, in ATC were explored as part of the BRF117019 (NCT02034110) trial. 16 patients with BRAF V600E mutant ATC were enrolled in this phase II, open-label trial. At a median follow-up of 47 weeks, ORR was 69% (11 of 16; 95% CI, 41–89%), and seven patients had continued response to therapy at the time of follow-up. Median DOR, PFS, and OS were not reached. The most common AEs seen were fatigue (38%), pyrexia (37%), and nausea (35%) (14).These data led to the FDA approval of dabrafenib and trametinib for BRAFV600E mutated ATC.

BRAF inhibition with dabrafenib or vemurafenib is also effective for DTC. In a non-randomized, open-label phase II study of vemurafenib in 51 patients with BRAF V600E mutated PTC, 10 of 26 patients who were VEGFR TKI-naive had PRs (38.5%, 95% CI 20.2–59.4) and a majority had at least SD (57.5%). Median PFS was 18.2 months (95% CI, 15.5–29.3 months). Median OS was not reached. Among 25 patients who had previously received a VEGFR TKI, 27.3% of patients had achieved a PR with 63.6% of patients achieving SD. Median PFS was only 8.9 months in comparison and OS was 14.4 months (95% CI, 8.2 to 29.5 months) (15).

In a phase II, randomized study, patients with BRAFV600E mutated PTC were randomized to dabrafenib or dabrafenib with trametinib. Among the 26 patients who received dabrafenib monotherapy, 10 of 26 (38%) had RECIST defined PR, while nine of 27 in the combination arm had a radiographic PR. A total of 50% and 54% in monotherapy and combination, respectively, had at least 20% decrease in target lesions. Median PFS was 11.4 months for dabrafenib and 15.1 months for dabrafinib and trametinib (19).

## Ret Inhibition

Selpercatinib, or LOXO-292, is an oral selective RET kinase inhibitor recently FDA-approved for RET mutated MTC and RET fusion-positive thyroid cancers. The phase I/II LIBRETTO-001 trial evaluated the safety and efficacy of selpercatinib in patients with RET-mutant MTC. Patients were treated with 160 mg of selpercatinib twice daily. Among 55 patients previously treated with TKIs including cabozantinib and vandetinib, ORR was 69% (95% CI; 55–81%). The median DOR was not reached at a median follow up of 14 months. Among 88 TKI naïve subjects, ORR was 73% (95% CI; 62–82%). Finally, in a cohort of patients with RET-fusion positive thyroid cancer, ORR was 62% (95% CI; 41–80%) with 16 patients with ongoing response and two PRs awaiting central confirmation. The most frequently reported adverse events were dry mouth (20), increased transaminases (25%), hypertension (24%), diarrhea (22%), fatigue (18%*)*, and peripheral edema (15%). The discontinuation rate due to side effects was only 2% (16).

Pralsetinib, BLU-667, is a second potent RET-inhibitor with activity in RET-fusion positive MTC and lung cancer. Among 13 RET-fusion positive TC patients enrolled in ARROW, a phase I/II trial of pralsetinib for RET-mutated cancers, ORR was 91% and all patients had stable disease or better. For RET-mutated treatment naïve MTC patients, ORR was74%, while for previously treated patients, a 60% ORR was reported (17).

## Mechanisms of Resistance and Treatment Strategies for TC

Patients with RAIR TC have a poor prognosis with a 10-year survival rate of only 10%. One mechanism underlying the development of RAIR thyroid cancer is impairment of the sodium-iodine symporter (NIS). NIS is a plasma membrane glycoprotein located on the basolateral surface of the thyroid follicular cells that mediates iodide transport into follicular cells. RAI enters TC cells *via* the NIS, and therefore, loss or downregulation of NIS through genetic alteration of the RTK/BRAF/MAPK/ERK and PI3K/AKT/mTOR pathways is thought to contribute to RAIR (5). Furthermore, constitutive activation of the MAPK pathway, and in particular, the presence of the BRAF activating mutation, alters genes involved in iodine metabolism, resulting in more aggressive tumorgenesis and thyroid cell de-differentiation. Consequently, BRAF V600E mutant TC have higher risk of relapse, poorer outcomes and are less likely to be responsive to RAI. Therefore, strategies to “re-sensitize” tumors to RAI utilizing BRAF and MEK inhibitors have been evaluated. 20 patients with RAIR TC were treated with MEK1/2 inhibitor selumetinib 75 mg twice daily (21). Following selumetinib treatment, RAI uptake increased in 12 of 20 patients. Eight of 12 patients were re-treated with RAI of which three achieved SD and five achieved PR. For BRAFV600E mutated PTC, dabrafenib treatment reinduced new RAI uptake in six of 10 patients, with two PRs and four SD at three months post RAI (22). This strategy has also been evaluated with vemurafenib in a pilot study of 12 BRAF mutated TC patients. Among the 10 evaluable patients, four demonstrated increased ^123^I uptake after four weeks of vemurafenib therapy. Treatment with ^131^I resulted in ongoing tumor control at 6 months. Of note, vemurafenib was discontinued two days after ^131^I treatment, raising the possibility of RAI treatment after MAPK inhibition as a strategy to allow for treatment breaks from TKIs, which can have significant toxicity (23).

## Braf Resistance

Several mechanisms can confer primary or secondary resistance to BRAF inhibitors (24). Intrinsic resistance to BRAF inhibitors may result from inhibition of apoptosis *via* inhibiting the B-Cell CLL/Lymphoma 2 (BCL2) pathway. *In vitro*, TC cells harboring a copy number gain of myeloid cell leukemia 1 (MCL1) as well as a loss of (cyclin-dependent kinase inhibitor 2A (CDK2NA), components of the BCL2 pathway, are resistant to vemurafenib, and combining the BCL2 inhibitor obatoclax with vemurafenib improved sensitivity (25). Furthermore, concurrent mutations in BRAFV600E and PI3KCA can confer intrinsic resistance to BRAF inhibitors (26). To this end, combination treatment with MAPK inhibitors and inhibitors targeting PI3K/AKT/mTOR pathway such as everolimus may be an effective strategy.

Indeed, everolimus does have some clinical activity for TC. In a phase II clinical trial, 28 patients with locally advanced or metastatic RAIR DTC and seven patients with ATC were treated with everolimus 10 mg daily. The median follow-up duration was 38 months. Seventeen patients (65%) had SD; however, no PR or CR was observed. The response was quite durable: 58% had SD for greater than 24 weeks. Toxicity was generally mild and consistent with its known side effect profile (27). Additionally, in a case series of five patients with ATC treated with everolimus 10mg daily, mOS was 7.4 months. One patient had a durable response that lasted 27.9 months, and two others had SD for 3.7 and 5.9 months, respectively (28).

Acquired resistance to BRAF inhibitors may develop *via* secondary mutations in the MAPK pathway, such as acquisition of NRAS Q61K (29, 30), or KRAS G12V (30), similar to that seen with BRAFV600E melanoma. Whether addition or substitution of MEK inhibitors, novel MAPK inhibitors such as KRAS or ERK inhibitors would be of clinical utility are intriguing potential treatment options. For VEGFR inhibitors such as lenvatinib, sorafenib, cabozantinib, and vandetinib, upregulation of FGFR may mediate acquired resistance, and anti-FGFR agents may have application in this setting (31).

## Combination Therapies

The utility of combining TKIs and immunotherapeutic agents is currently an area of active investigation. Several published case series in TC highlight the potential for combination targeted therapy with checkpoint inhibition to confer prolonged tumor control, even in patients who have progressed on prior targeted therapy (32, 33). Eight patients with metastatic ATC or DTC were treated with lenvatinib (24 mg/kg daily) in combination with pembrolizumab (200 mg every 3 weeks). Four patients achieved a PR and two achieved SD. One patient achieved a CR and one patient expired from PD. Notably, no significant grade 3 or 4 toxicities were observed with treatment (20). Among 30 patients with VEGFR-inhibitor naïve progressive DTC, 18 of 30 patients (62%) treated with lenvatinib and pembrolizumab had a PR, and 10 (35%) had SD (34). A separate cohort of this study is evaluating responses among patient who have progressed on lenvatinib (NCT02973997). For ATC, in a single institution study of 38 patients, the combination of atezolizumab with vemurafenib and cobimetinib for BRAF V600E mutant demonstrated an ORR of 59%, including one CR and 9 PR, and seven SD. An 81 and 70% 1- and 2-year survival, respectively, with median OS were not reached. For the ATC cohort with RAS or NF1 or NF2 alterations, patients were treated with cobimetinib and atezolimab with an ORR 17%, with 2 PR and four SD (35). Ongoing clinical trials for RAIR TC are listed in **Table 2**.

**Table 2:** Ongoing Clinical trials for Radioactive Iodine Refractory Thyroid Cancer.

Intervention/Treatment	Phase	Disease Type	Trial Name/Number
Lenvatinib + Denosumab	2	Metastatic DTC	NCT03732495
Cyclophosphamide + Sirolimus	2	Metastatic DTC	NCT03099356
RAI + Durvalumab	1	RAI avid/M TC	NCT03215095
177Lu-PP-F11N + Sacuitril	1	Metastatic MTC	NCT03647657
RAI + Selumetinib	2	RAI avid R/M TC	NCT02393690
RAI + Trametinib	2	RAI refractory R/M TC	NCT02152995
Durvalumab + Tremelimumab	2	Metastatic DTC	NCT03753919
177Lu-PP-F11N	1	Metastatic MTC	NCT02088645
Imatinib	1	PTC	NCT03469011
Durvalumab + Tremelimumab	1	Metastatic ATC	NCT03122496
RAI + Dabrafenib/Trametinib	2	RAIR R/M TC +RAS/BRAF mutated	NCT03244956
Regorafenib	2	Metastatic MTC	NCT02657551
MLN0128	2	Metastatic ATC	NCT02244463
Apatinib Mesylate	2	Locally Advanced or Metastatic DTC	NCT03167385
Dabrafenib + Lapatinib	1	Unresectable or Metastatic TC	NCT01947023
Apatinib	2	Advanced and Metastatic DTC	NCT04180007
Sorafenib Tosylate	2	Locally Advanced or R/M MTC	NCT00390325
Apatinib	2	RAI refractory Locally Advanced or R/M DTC	NCT02731352
Apatinib	3	RAI refractory DTC	NCT03048877
Pembroliumab	2	Undifferentiated/ATC	NCT02688608
Vandetinib	3	Locally Advanced or Metastatic DTC	NCT01876784
Vandetinib	3	Unresectable Locally Advanced or Metastatic MTC	NCT00410761
Bevacizumab + Temsirolimus +/− Valproic Acid or Cetuximab	1	R/M TC	NCT01552434
TPX-0046	1/2	RET Fusion or Mutated Solid Tumors	NCT04161391
Lenvatinib + pembrolizumab (34)	2	DTC	NCT02973997
Atezolizumab + Chemotherapy (35)	2	Undifferentiated/ATC	NCT03181100

DTC, differentiated thyroid carcinoma; RAI, radioactive iodine; M, metastatic; TC, thyroid cancer; MTC, medullary thyroid carcinoma; R/M, recurrent/metastatic; ATC, anaplastic thyroid carcinoma.

## Conclusions

There are currently a number of therapies available for treatment of metastatic TC. Understanding the molecular mechanisms underlying pathogenesis of thyroid malignancies has allowed the development of a myriad of effective therapies targeting these underlying mechanisms. Most successful has been the use of TKIs which can portend improvement in PFS, and even OS, in the case of lenvatinib. Targeting BRAF mutations as well as inhibition of RET has led to further approvals for treatments in this space. Nonetheless, many patients develop resistance to these therapies, and therefore the focus on developing newer, more effective treatments has become even more pressing.

## Author Contributions

AP and DW contributed equally to the conception and design of the manuscript and in the drafting and critical revision of the manuscript. All authors contributed to the article and approved the submitted version.

## Conflict of Interest

The authors declare that the research was conducted in the absence of any commercial or financial relationships that could be construed as a potential conflict of interest.
